# Leg Cycling Leads to Improvement of Spasticity by Enhancement of Presynaptic Inhibition in Patients with Cerebral Palsy

**DOI:** 10.1298/ptr.E10228

**Published:** 2023-06-20

**Authors:** Senshu ABE, Yuichiro YOKOI, Naoki KOZUKA

**Affiliations:** ^1^Department of Physical Therapy, Rehabilitation Part, Hokuto Social Medical Corporation, Tokachi Rehabilitation Center, Japan; ^2^Advanced Rehabilitation Office, Hokuto Social Medical Corporation, Tokachi Rehabilitation Center, Japan; ^3^Department of Physical Therapy, Rehabilitation of Healthcare and Science, Hokkaido Bunkyo University, Japan; ^4^Department of Physical Therapy, School of Health Sciences, Sapporo Medical University, Japan

**Keywords:** Cerebral palsy, Spasticity, Pendulum test, Cycling, Presynaptic inhibition

## Abstract

Objective: The purpose of this study was to investigate if leg cycling could reduce lower extremity spasticity in patients with cerebral palsy (CP). In addition, we investigated whether the intervention could cause changes in the modulation of presynaptic inhibition. Methods: This study was a quasi-experimental study, with pretest–posttest for 1 group. Participants in this experiment were eight adult patients with CP with lower extremity spasticity. Spasticity parameters assessed were the amplitude of soleus maximum Hoffmann’s reflex (H_max_) and maximum angular velocity (MAV) of knee flexion measured using the pendulum test. D1 inhibition, which seems to be related to the presynaptic inhibition, was recorded by measuring soleus Hoffmann’s reflex (H-reflex) with conditioned electric stimuli to the common peroneal nerve. Results: D1 inhibition was significantly enhanced immediately by the cycling intervention. The amplitude of the soleus H_max_ was significantly depressed, and there was significant difference in H_max_/maximum M-wave. The MAV was increased due to inhibition of the stretch reflex. Conclusion: Leg cycling suppressed stretch reflex and H-reflex, and caused plasticity of inhibitory circuits in patients with CP with lower extremity spasticity. These findings strongly suggest that lower extremity spasticity can be improved by cycling movements.

**C**erebral palsy (CP) includes a group of permanent disorders of movement and postural development that cause activity limitations and can be attributed to non-progressive disturbances in the developing fetal or infant brain^1)^. The motor disorders associated with CP are often accompanied by secondary musculoskeletal problems^2)^. Spasticity is defined as a motor disorder characterized by velocity-dependent stretch reflexes with exaggerated tendon jerks, which is a symptom of upper motor neuron syndrome^3)^. Spastic CP accounts for approximately 80%–90% of all patients with CP^4)^; they suffer from symptoms such as muscle stiffness^5)^, shortening, and increase risk for falls^6)^. Moreover, the contracture responsible for stiffness causes further muscle overactivity, including spasticity^7)^. Although spasticity has been attributed to lesions of the pyramidal tract, in animals, isolated lesions of the primary motor cortex (Broadman area 4) have been shown to decrease muscle tone and tendon reflexes instead of causing spasticity. Moreover, lesions of the premotor cortex and supplementary motor cortex (Broadman area 6) have been often shown to induce spasticity^8^^,^^9)^. Even though pathophysiological mechanisms underlying spasticity in humans are not completely understood, the evidence suggests that an indirect descending pathway modulating the stretch reflex circuit from the motor cortex, such as the vestibulospinal tract, and presynaptic Ia inhibition is one of the spinal inhibitory interneuron systems involved in stretch reflex modulation^10^^,^^11)^. D1 inhibition^12)^, which seems to be related to the excitability of primary afferent depolarization interneurons mediating presynaptic inhibition of Ia terminals, deteriorates in CP^13)^. In addition, several studies have reported that patients with stroke^14)^ and multiple sclerosis^15)^ show increased Hoffmann’s reflex (H-reflex) amplitude and impaired presynaptic Ia inhibition in the lower limbs in comparison with healthy controls.

Considering these findings, appropriate interventions for spasticity are extremely important, and its management is currently achieved through various therapeutic exercises, pharmacotherapy, and orthopedic surgery. In a recent systematic review on the evidence for interventions for CP, botulinum toxin, diazepam, intrathecal baclofen, and selective dorsal rhizotomy were recommended for reducing muscle spasticity^16)^, while no established evidence could be identified for therapeutic exercise. Nevertheless, we have experienced decreased muscle tone in patients with CP after cycling exercise in our daily clinical practice. Recent investigations have demonstrated that cycling movements induce a reduction in spasticity variables such as the H-reflex amplitude and Modified Ashworth Scale (MAS) scores^17)^ and enhance inhibitory spinal pathways such as those mediating presynaptic inhibition in patients with diseases of the central nervous system other than CP^18^^–^^21)^. In contrast, only a few studies have focused on the combination of electrophysiological measurements and use of this therapeutic intervention for spasticity in CP, and its effects on the modulation of spinal inhibitory interneuron systems in patients with spastic CP.

Rhythmic movements such as cycling and walking have different neural controls than normal voluntary movements^22^^,^^23)^. The common core hypothesis suggests the existence of central pattern generators (CPGs) and regulatory systems that modulate reflex output within the spinal cord. The reason for focusing on D1 inhibition is that presynaptic inhibition may contribute to inputs from higher centers, CPGs, and even peripheral sensory receptors to modulate reflex outputs in the spinal cord.

Moreover, the spasticity in CP was often evaluated using the MAS according to published literature and in clinical practice, although some studies have questioned the reliability and validity of this evaluation^24^^–^^26)^, and little evidence is available for the quantitative evaluation of spasticity. In this regard, the pendulum test^27)^, which is performed by freely dropping the lower leg from knee extension in a relaxed state, has proven to be a reliable and objective method to assess various aspects of spasticity in CP^28^^,^^29)^.

This study aimed to investigate if leg cycling could reduce lower extremity spasticity in patients with CP. In addition, we investigated whether the modulation of presynaptic inhibition (i.e., D1 inhibition) changed after this intervention.

## Methods

### Study design

This study was a non-randomized, quasi-experimental study, with pretest–posttest for 1 group.

### Participants

Fourteen adult patients with CP were recruited from Sapporo Medical University Hospital. Six of them were unable to start the electrophysiological measurement protocol because of discomfort and excessive involuntary movements in response to the slight stimulus. The remaining eight patients (mean age, 33.4 years; standard deviation, 11.3 years; range 19–45 years; seven males and one female in each year) finally participated in this study. The functional level of participants was classified according to the Gross Motor Function Classification System^30)^ (GMFCS). Six patients with CP were classified as level I and two were classified as level III. CP subtypes were also assigned by classifying four patients with spastic diplegia, one with hemiplegia, and three with mixed CP. While kinematic measurement was feasible for all participants, electrophysiological measurements showed that so while D1 inhibition was evaluable in seven patients, the maximum M wave (M_max_) in the soleus muscle was obtained from five participants because three of them could not tolerate the maximal supra-stimulus intensity to the nerve. The study protocol was approved by Sapporo Medical University Hospital Institutional Review Board (Permit Number: 282-32) and was conducted in accordance with the Declaration of Helsinki of 1975, as revised in 2013. Written informed consent was obtained from patients who participated in this study.

Inclusion criteria were: (1) adults with CP and lower extremity spasticity (quadriceps and soleus muscle MAS score ≥1), (2) ability to comply with simple verbal directions, (3) GMFCS levels I to III, (4) ability to pedal the ergometer, and (5) knee extension range of motion ≥–30°.

Exclusion criteria were: (1) orthopedic surgery within six months preceding the experiment, (2) a history of botulinum injections or other medications used in the treatment of spasticity within three months preceding the experiment, and (3) difficulty in cycling without orthoses.

Electrophysiological and kinematic measurements were obtained using the H-reflex method and pendulum test, respectively. Each measurement was obtained only for the more affected spastic lower extremity.

### Electrophysiological measurements

The participants were required to take a break of 10 min before the experiment, subsequently seated comfortably in a wheelchair with the knees flexed approximately 70° and the ankle joint fixed in the mid position. Electromyographic (EMG) activities were recorded using bipolar surface electrodes (NM-317Y3; Nihon Kohden, Tokyo, Japan) placed 2 cm apart over the muscle bellies of the soleus and tibialis anterior (two-thirds of the distance between the medial condyle of the femur and the medial malleolus, and the proximal one-third of the distance between the caput fibulae and the medial malleolus).

The EMG signals were amplified and sampled at 2000 Hz, and band-pass filtered at 10–1000 Hz, capable of making measurements across a ±5 mV range (10 mV span) using an 16-bit A/D converter connected to a computer (Neuropack MEB-2306; Nihon Kohden). Soleus H-reflexes were elicited by percutaneous stimulation (rectangular pulse of 1 ms duration, 0.33 Hz) of the posterior tibial nerve in the popliteal fossa through the bipolar stimulating electrode. D1 inhibition was evoked by soleus H-reflex induced with an electrical stimulus applied to the common peroneal nerve (CPN)^12)^.

**Fig. 1.**
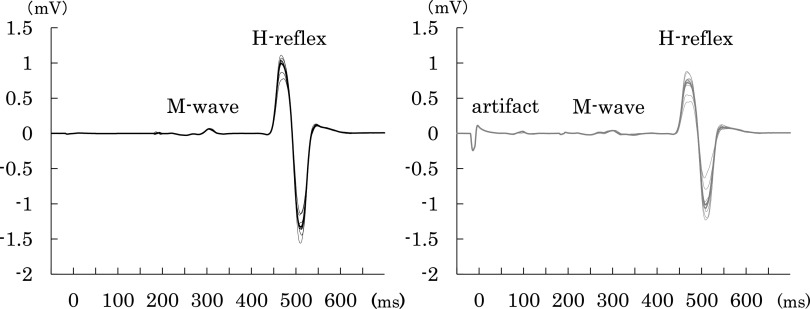
The control H-reflex (black line) is adjusted to 50% of H_max_

The conditioning stimulus was generated at the dorsolateral aspect of the caput fibulae with a rectangular pulse of 1 ms duration and an intensity that was 1.2 fold the motor threshold, and it preceded the test stimulus with a 20-ms interstimulus interval (ISI). The control H-reflex intensity was adjusted at 50% of the ipsilateral soleus maximum H-reflex (H_max_), and both conditioned and control H-reflexes were recorded in 10 sweeps each (Fig. 1). Conditioning stimulation to the CPN was performed while monitoring tibialis anterior EMG and H-reflex to ensure consistency of stimulation. D1 inhibition was indicated by the following formula: D1 inhibition (%) = (conditioned-H reflex amplitude/control-H reflex amplitude) × 100; a decrease in this value indicates suppression conditioned H-reflex due to the potentiation of presynaptic Ia inhibition. The H-reflexes and M-waves were expressed as peak-to-peak amplitudes, and the ratio of the H_max_ to M_max_ (H_max_/M_max_) was determined in the recruitment curve of each participant.

The conditioned H-reflex (gray line) is expressed as suppression of the soleus H-reflex with conditioning stimulus. Depicted waveforms are superimposed (thin line) and average (bold line) waves of 10 sweeps for each of the reflexes.

H-reflex, Hoffmann’s reflex; H_max_, maximum H-reflex

### Kinematic measurements

The pendulum test was performed with the patient semi-reclined comfortably on the medical bed with legs hanging freely over the edge. A wireless gyro sensor (WAA-006; Wireless Technology, Tokyo, Japan) was attached to the front of lower leg distal to the side tested, and the swinging motion with angular velocity of the rotation around the X axis was recorded and sampled at 250 Hz. The examiner passively lifted the lower limb to full extension and held the leg position until the state of complete relaxation for 5 s or more, as indicated by palpation and inspection of quadriceps contraction and spasm. Subsequently, the relaxed limb was released to swing freely and allowed to oscillate until it stopped at a resting position. The maximum angular velocity (MAV) during the first swing, which was obtained using the gyro sensor, was stored on a computer for subsequent offline analysis. As for data processing, zero point correction was performed on angular velocity from data in which the sensor was stationary for 3 s for each patient.

### Interventions

All participants performed a 20-min exercise at a 20-W constant work rate on the ergometer. Adjustable foot straps and magic tape were used to hold the participants’ feet and prevent slipping. The saddle height was adjusted so that the knee flexion angle was approximately 20°–30° at the bottom dead center of the pedal. The participants were instructed to cycle at a comfortable rate with smooth movements without clonus and stretch reflexes. Therefore, we did not control the rotation speed for each participant, although made sure that it was not less than approximately 20 rpm. The positions of the surface electrodes and accelerometer were ensured to remain constant during the intervention.

### Statistical analyses

Statistical analyses were performed using the Statistical Package for the Social Sciences version 21 (IBM, Armonk, NY, USA). The results were expressed as mean ± standard error of the mean. Differences between the measurements before and after the intervention were calculated with a paired two-tailed t-test. The effect size was calculated by Cohen’s d^31)^ for only parametric tests (Cohen’s operational definitions; d ≥0.20, d ≥0.50, and d ≥0.80 for small, medium, and large effect sizes, respectively). Statistical significance was determined by a p-value of 0.05.

## Results

**Fig. 2.**
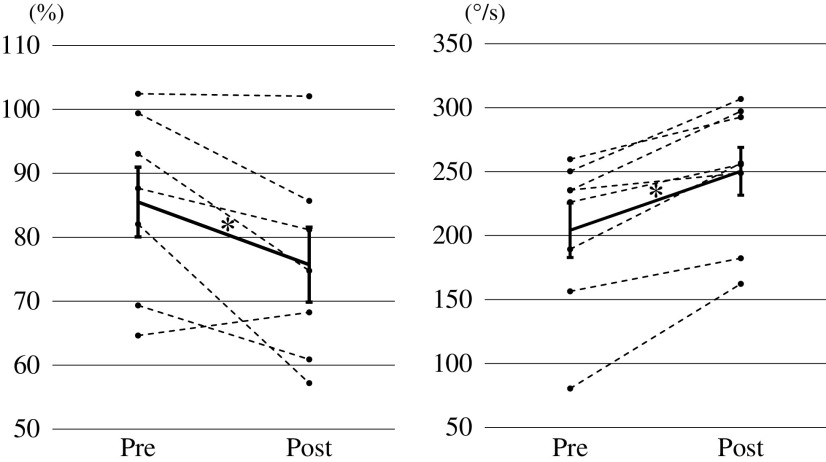
Changes in D1 inhibition (left figure) and MAV of knee flexion (right figure) in the pendulum test

The results are shown in Table 1. D1 inhibition was significantly enhanced after the cycling intervention (pre: 85.51 ± 5.45%, post: 75.72 ± 5.86%, t = 2.602, p = 0.041; Fig. 2, left). The amplitudes of the soleus H_max_ were significantly depressed (pre: 2.58 ± 0.49 mV, post: 2.05 ± 0.53 mV, t = 8.454, p = 0.001), but no significant differences were observed in M_max_ before and after the intervention (pre: 6.83 ± 1.10 mV, post: 7.03 ± 1.12 mV, t = 0.881, p = 0.428), and so there was a significant reduction in H_max_/M_max_ ratio (pre: 0.385 ± 0.065, post: 0.282 ± 0.050, t = 4.008, p = 0.016). In addition, the MAV values significantly increased after the intervention (pre: 204.1 ± 21.3°/s, post: 250.3 ± 18.7°/s, t = 5.445, p = 0.001; Fig. 2, right).

**Table 1.** Descriptive statistics of all variables calculated from soleus H-reflex and the pendulum test

	Pre	Post	Effect size	p-value
D1 inhibition (%)	85.51 ± 5.45	75.72 ± 5.86	d = 0.654	p = 0.041*
H_max_ (mV)	2.58 ± 0.49	2.05 ± 0.53	d = 0.465	p = 0.001*
M_max_ (mV)	6.83 ± 1.10	7.03 ± 1.12	d = 0.080	p = 0.428
H_max_/M_max_	0.385 ± 0.065	0.282 ± 0.050	d = 0.792	p = 0.016*
MAV (deg/s)	204.1 ± 21.3	250.3 ± 18.7	d = 0.815	p = 0.001*

Data are expressed as mean ± standard error of the mean. Asterisks indicate significant differences (p <0.05).

H-reflex, Hoffmann’s reflex; H_max_, maximum H-reflex; M_max_, maximum M-wave; MAV, maximum angular velocity

The mean and standard error of the mean values are presented in bold lines and circles, and the dashed lines indicate changes in the values of each individual. Asterisks indicate significant differences (p <0.05).

MAV, maximum angular velocity

## Discussion

In a series of experiments in patients with CP, the present study showed that leg cycling caused (i) enhancement of D1 inhibition, (ii) suppression of H-reflex amplitude, and (iii) increase in MAV.

The currently accepted hypothesis of spasticity focuses on inhibitory rather than excitatory mechanisms in the spinal circuit^10)^. An increase in D1 inhibition reflects the enhancement of presynaptic Ia inhibition, which is one of the inhibitory mechanisms in spinal neural circuits that are impaired in patients with spasticity^13^^–^^15)^. In a previous study, the mean suppression of the H-reflex by D1 inhibition was 86.19% on the paralyzed side and 77.64% on the non- paralyzed side in stroke patients with spasticity (n = 29)^13)^, 87.56% in children with CP (n = 21), and 69.56% (n = 21) in non-disabled children^14)^. These findings are similar to the pre- and post-intervention suppression in the present study. Long-term cycling exercises in patients with spinocerebellar ataxia causing coordination defects have been shown to enhance D1 inhibition concurrently with improvements in ataxia score^32)^. Thus, the enhancement of D1 inhibition may be related to agonist and antagonist control in the cycling task. Spastic CP is characterized by impairments in coordination and selective motor control in the lower extremities, particularly the distal joints^1^^,^^33)^. Patients with spastic CP show high co-contraction ratios for the lower limb muscles during a single cycling session compared with healthy participants^34)^. Whether an improvement in selective motor control of the lower extremities leads to enhanced D1 inhibition is a topic for future studies.

Another possibility is that increases in presynaptic inhibition by rhythmic and reciprocal movements influence several spinal pathways and afferent fibers. Numerous studies have demonstrated that the H-reflex of the lower limb changes during and after lower extremity cycling exercise in a task-dependent manner^18^^,^^19^^,^^35^^,^^36)^. Furthermore, other studies have reported that the soleus H-reflex decreases after arm cycling exercise^19)^. When it was conditioned with CPN stimulation during arm cycling^37)^, a greater reduction than that in static control was observed. These findings support the role of excitability of presynaptic inhibition in the activation of supraspinal and propriospinal pathways and the CPGs in cyclic arm movement. Neural mechanisms such as the CPGs and peripheral feedback during rhythmic movement have specific modulatory effects on reflex inhibition^38)^. The increased presynaptic inhibition in cycling probably contributed to the degradation of the input of Ia afferent fibers and suppressed the soleus H-reflex; additionally, D1 inhibition was enhanced in this study. Previous studies have shown that the soleus H-reflex is depressed by continuous passive and active cycling for at least 30 min. Since passive cycling involves almost no muscle contraction, afferent input from types III and IV afferent mechanoreceptors may be associated with presynaptic inhibition of Ia afferent terminals and contributes to reflex control^35^^,^^39)^.

These findings suggest that downregulation of the H-reflex is less related to descending motor pathways such as corticospinal or corticobulbar tracts and is more associated with increased fusimotor drive, Ia activity, and primary afferent depolarization^40)^. Post-activation depression^41)^, one of the presynaptic mechanisms underlying spasticity, is related to muscle spasticity^13^^,^^14)^. However, a previous study has reported that inhibition of spasticity is not explained by its depression since cycling training decreased the soleus H-reflex and MAS score for calf muscle in contrast to the sustained post-activation depression observed in patients with multiple sclerosis^19)^. Thus, H-reflex suppression after cycling exercise in patients with CP may have been caused by the modulation of presynaptic inhibition rather than post-activation depression.

The increased MAV indicated that the velocity-dependent stretch reflex in the quadriceps was inhibited by this intervention. The MAV (mean ± standard deviation) for children with (n = 10) and without (n = 10) CP in a previous report was 201.82 ± 67.96°/s and 292.51 ± 35.93°/s, respectively, suggesting that MAV is significantly lower in children with CP^28)^. Another study found that among 20 children with CP, the mean MAV improved from 244°/s to 364°/s after selective dorsal rhizotomy to control spasticity^29)^. In our study, the increase in the MAV was statistically significant, although it was not close to this value.

Suppression of the soleus H-reflex and enhancement of D1 inhibition may be the result of a specific effect of pedaling exercise. Previous studies have described task-dependent changes in the soleus H-reflex during and after pedaling in able-bodied people^35^^,^^36)^. In the present study, the stretch reflex of the quadriceps was suppressed. While there are no studies assessing quadriceps stretch reflexes after pedaling, studies report decreased hamstring MAS^42)^. Since pedaling generates muscle activity in the quadriceps, hamstrings, and lower leg in CP^34)^, it is possible that the suppression of the reflex occurred in a task-dependent manner not only in the soleus muscle but also in the quadriceps.

The suppressed H-reflex in D1 inhibition could not be confirmed in the hemiplegic patient (pre: 102.4%, post: 102.1% of control H-reflex). Strongly impaired D1 inhibition in spastic hemiplegic CP has been previously reported, but the number of participants was small^13)^ (N = 3, 99.14 ± 6.58% of control H-reflex). In contrast, another study found that spastic stroke patients have impaired D1 inhibition compared to healthy participants^14)^. Regarding the amount of H-reflex suppression, it is possible that stroke patients have a smaller degree of D1 inhibition of the damage despite the shorter time from onset than hemiplegic CP.

We hypothesized that impaired H-reflex inhibition in hemiplegic patients with CP may be related to the timing of brain damage in infancy or fetal life because GABAergic neurons in the central nervous system have been shown to change from excitatory to inhibitory synapses during early neuronal development in animal testing^43^^,^^44)^. In the participants with hemiplegia in our study, D1 inhibition was not modified, whereas the reflex (i.e., H-reflex and MAV) was inhibited. Hence, the exaggerated reflex in hemiplegic CP must be affected by mechanisms other than presynaptic inhibition, but further data accumulation is necessary.

The clinical significance of these results is that the enhancement of the spinal inhibitory interneuron is a result of reciprocal movement. Moreover, the cycling task is easy to use in clinical practice and can be recommended as an exercise therapy. For future studies, the effect on motor functions such as walking speed and endurance, as well as the long-term effects and required duration of intervention need to be further examined. The intervention time in this study was within the same time frame as that of several previous studies, and similar results were obtained. However, there are reports of changes in spasticity scores in even shorter time periods.

The present study has some limitations. The ISI required to obtain maximum suppression of the H-reflex in D1 inhibition is approximately 20 ms, and many studies have used 20 or 21 ms^13^^,^^14^^,^^32)^. However, it is important to note that this value may vary among individuals^12)^. Moreover, this experiment was performed with participants showing several types of CP (e.g., hemiplegia, diplegia, and mixed), a wide range of ages, and a relatively small sample size, which could limit the interpretation of the results. Nevertheless, we anticipate that cycling movements will become an effective intervention for the treatment of spasticity in patients with CP.

## Conclusion

The findings of the present study suggest that cycling intervention in adults with CP can induce suppression of H-reflex and stretch reflex in the leg, and activation of presynaptic inhibitory networks within the spinal cord. Therefore, leg cycling may reduce spasticity in the lower extremities of patients with CP.

## Acknowledgments

We are grateful to Dr. Megumi Toki, Assistant Professor of Sapporo Medical University, for her important contributions to the experiments. This study was supported by the Japanese Physical Therapy Association (JPTA H28-B39).

## Conflict of Interest

The authors have no conflicts of interest in relation to this manuscript.
